# Gold nanoparticles administration induces disarray of heart muscle, hemorrhagic, chronic inflammatory cells infiltrated by small lymphocytes, cytoplasmic vacuolization and congested and dilated blood vessels

**DOI:** 10.1186/1476-511X-10-233

**Published:** 2011-12-09

**Authors:** Mohamed Anwar K Abdelhalim

**Affiliations:** 1 Department of Physics and Astronomy, College of Science, King Saud University, Saudi Arabia

**Keywords:** gold nanoparticles, size, heart muscle, histology, inflammatory, nanotoxicity, cytoplasmic vacuolization, rats

## Abstract

**Background:**

Despite significant research efforts on cancer therapy, diagnostics and imaging, many challenges remain unsolved. There are many unknown details regarding the interaction of nanoparticles (NPs) and biological systems. The structure and properties of gold nanoparticles (GNPs) make them useful for a wide array of biological applications. However, for the application of GNPs in therapy and drug delivery, knowledge regarding their bioaccumulation and associated local or systemic toxicity is necessary. Information on the biological fate of NPs, including distribution, accumulation, metabolism, and organ specific toxicity is still minimal. Studies specifically dealing with the toxicity of NPs are rare. The aim of the present study was to investigate the effects of intraperitoneal administration of GNPs on histological alterations of the heart tissue of rats in an attempt to identify and understand the toxicity and the potential role of GNPs as a therapeutic and diagnostic tool.

**Methods:**

A total of 40 healthy male Wistar-Kyoto rats received 50 μl infusions of 10, 20 and 50 nm GNPs for 3 or 7 days. Animals were randomly divided into groups: 6 GNP-treated rats groups and one control group (NG). Groups 1, 2 and 3 received infusions of 50 μl GNPs of size 10 nm (3 or 7 days), 20 nm (3 or 7 days) and 50 nm (3 or 7 days), respectively.

**Results:**

In comparison with the respective control rats, exposure to GNPs doses produced heart muscle disarray with a few scattered chronic inflammatory cells infiltrated by small lymphocytes, foci of hemorrhage with extravasation of red blood cells, some scattered cytoplasmic vacuolization and congested and dilated blood vessels. None of the above alterations were observed in the heart muscle of any member of the control group.

**Conclusions:**

The alterations induced by intraperitoneal administration of GNPs were size-dependent, with smaller ones inducing greater affects, and were also related to the time exposure to GNPs. These alterations may indicate scattered cytoplasmic vacuolization, which may induce the toxicity effect through an inability to deal with the accumulated residues resulting from metabolic and structural disturbances caused by these NPs. These histological alterations were more prominent with 10 nm size particles than with the larger ones. The interaction of GNPs with proteins and various cell types should be considered as part of the toxicological evaluation. Additional experiments related to plasma, tissues cytokine, antioxidant defense mechanism, lipid peroxidation, histomorphologcal and ultrastructure will be performed to identify and understand the toxicity and the potential use of GNPs as therapeutic and diagnostic tools.

## Introduction

Nanoparticles (NPs) are being investigated for gene delivery purposes [1-3] and cancer therapy [4]. Data concerning the behavior and toxicity of particles mainly comes from studies on inhaled NPs [5].

NPs may differ in reactivity and solubility and may interact with various endogenous proteins, lipids, polysaccharides and cells. Based on experiences in inhalation toxicology, a series of tests was proposed for evaluation of the toxicity of nanoparticles used in drug delivery systems [6]. GNPs can easily enter cells, and the demonstration that amine and thiol groups bind strongly to GNPs has enabled their surface modification with amino acids and proteins for biomedical applications [7,8].

All NPs, upon exposure to tissues and fluids of the body, will immediately adsorb some of the macromolecules that they encounter at their portal of entry onto their surface. The specific features of this adsorption process will depend on the surface characteristics of the particles, including surface chemistry and surface energy, and may be modulated by intentional modification or functionalization of the surfaces [9].

Gold, in its bulk form, has been considered an inert, noble metal with therapeutic and medicinal value. Gold nanoparticles (GNPs) are thought also to be relatively non-cytotoxic [10], while the metallic nature of metal-derived NPs and the presence of transition metals encourages the production of reactive oxygen species (ROS), leading to oxidative stress [11,12]. The use of nanoparticles as drug carriers may reduce the toxicity of the incorporated drug (Kim et al 2003). There are differing reports on the extent of the toxicity of these particles due to the variety of GNP modifications, surface functional attachments and shape and diameter size of the NPs [13,14].

The particle size-dependent organ distribution of GNPs has been studied in vivo [15-17]. In vivo studies in rats exposed to aerosols of GNPs revealed that NPs were rapidly taken into the system, with the highest accumulation in the lungs, aorta, esophagus and olfactory bulb [18].

To understand and categorize the mechanisms behind NP toxicity, information is needed on the response of living systems to the presence of NPs of varying size, shape, surface and bulk chemical composition. Very little information on these aspects is presently available and this implies that there is an urgent need for toxicokinetic data for NPs.

The histological and histochemical characterization of the heart tissues in the presence of GNPs has not been documented. In the present study, we aimed to characterize the possible histological alterations in the heart tissues after intraperitoneal administration of GNPs and, if alterations do occur, to identify whether they are related to the size of the GNPs and the time of exposure.

## Materials and methods

A total of 40 healthy male Wistar-Kyoto rats were obtained from the Laboratory Animal Center (College of Pharmacy, King Saud University, Saudi Arabia). The rats were nearly of uniform age (12 weeks) and weighed 220-240 g. Animals were randomly divided into groups, 3 GNP-treated rat groups and one control group (CG). 10, 20 and 50 nm GNPs were administered intraperitonealy at a rate of 3 or 7 days as follows: Group 1 received an infusion of 50 μl GNPs of size 10 nm for 3 or 7 days (n = 10); Group 2 received an infusion of 50 μl GNPs of size 20 nm for 3 or 7 days (n = 10); Group 3 received an infusion of 50 μl GNPs of size 50 nm for 3 or 7 days (n = 10). The control group received no GNPs (n = 10).

The rats were maintained on standard laboratory rodent diet pellets and housed in humidity and temperature-controlled ventilated cages on a 12 h day/night cycle. All experiments were conducted in accordance with the guidelines approved by King Saud University Local Animal Care and Use Committee.

Fresh portions of the heart from each rat were cut rapidly, fixed in neutral buffered formalin (10%), then dehydrated, with sequential exposure to grades of ethanol (70, 80, 90, 95 and 100%). Dehydration was followed by clearing the samples in 2 changes of xylene. Samples were then impregnated with 2 changes of molten paraffin wax, then embedded and blocked out. Paraffin sections (4-5 μm) were stained with haematoxylin and eosin (a conventional histological stain) according to Pearse [19]. Bright-field images were acquired using a Nikon Eclipse 800 microscope equipped with a Nikon DXM1200 color CCD camera (Nikon Instruments Inc., Melville, NY).

Stained sections of control and GNPs-treated rats were examined for alterations in the heart tissues.

## Results and discussion

### Size and morphology of different gold nanoparticles (GNPs)

The 10 and 20 nm GNPs had a spherical shape, while the 50 nm GNPs were hexagonal. The mean size of GNPs was calculated from images taken by the transmission electron microscope (TEM): The 10 nm GNPs had a mean size 9.45 ± 1.33 nm, 20 nm GNPs had a mean size 20.18 ± 1.80 and 50 nm GNPs had a mean size 50.73 ± 3.58 [20-23].

### Histological alterations

No mortality occurred during the GNP administration periods of 3 and 7 days in any of the experimental groups of the present investigation, and no alterations were observed in the appearance and behavior of GNP-treated rats in comparison with the control group.

**Figure 1:**
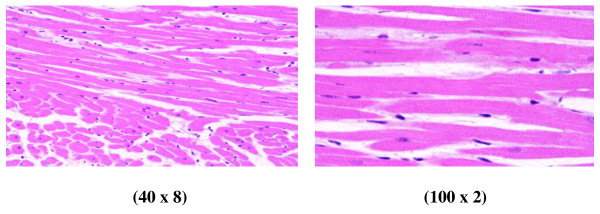
**GNP-normal rat demonstrating normal heart muscle**.

Control group (Figure 1): Microscopic pictures show GNPs-normal rat demonstrating benign, blunt-looking heart muscle with various heart muscle orientations and with no pathological findings.

**Figure 2:**
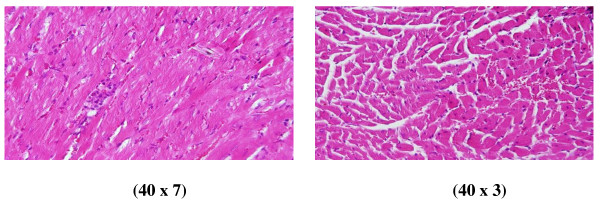
**GNP-treated rat that received 50 μl of 10 nm particles for 3 days demonstrating extravasation of red blood cells with a few scattered lymphocytic infiltrations**.

**Figure 3:**
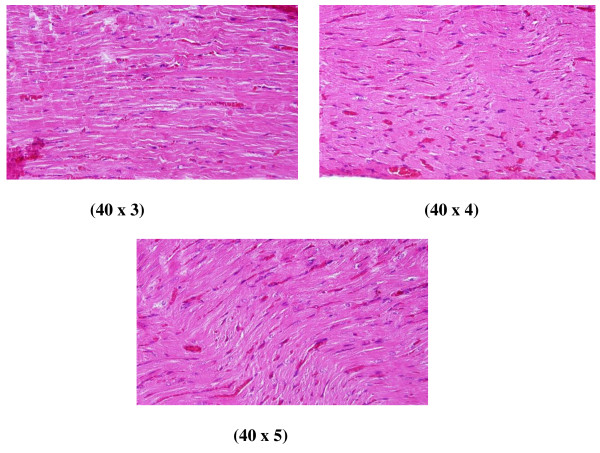
**GNP-treated rat that received 50 μl of 10 nm particles for 7 days demonstrating more dense hemorrhage and excess extravasation of red blood cells**.

**Figure 4:**
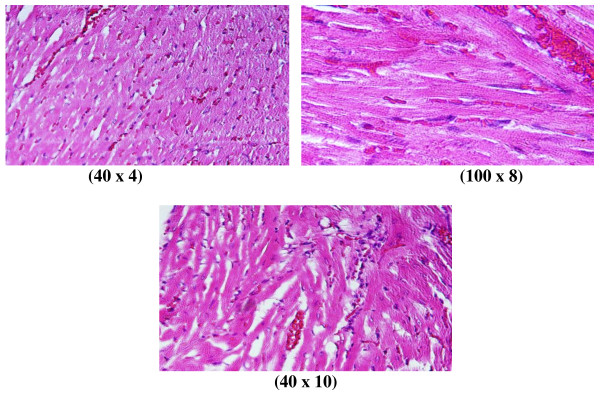
**GNP-treated rat that received 50 μl of 20 nm particles for 3 days demonstrating scattered extravasation of red blood cells associated with scattered to collected groups of lymphocytic infiltrations**.

**Figure 5:**
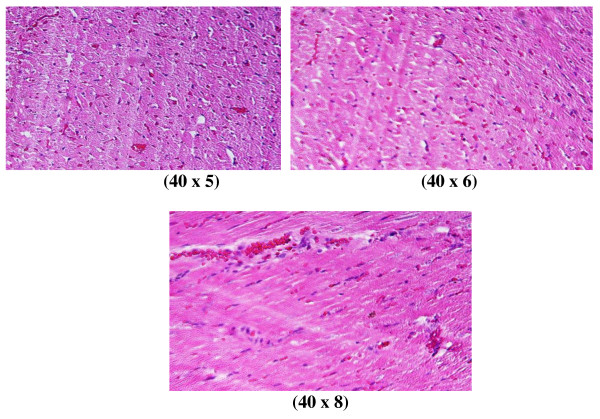
**GNP-treated rat that received 50 μl of 20 nm particles for 7 days demonstrating extravasation of red blood cells with a few scattered lymphocytic infiltrations**.

**Figure 6:**
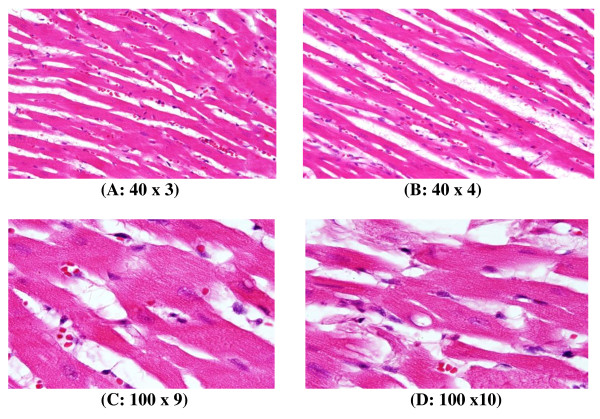
**GNP-treated rat that received 50 μl of 50 nm particles for 3 days; A and B demonstrate scattered foci of hemorrhage with extravasation red blood cells; C and D demonstrate a some scattered cytoplasmic vacuolization, which may indicate a toxicity effect**.

**Figure 7:**
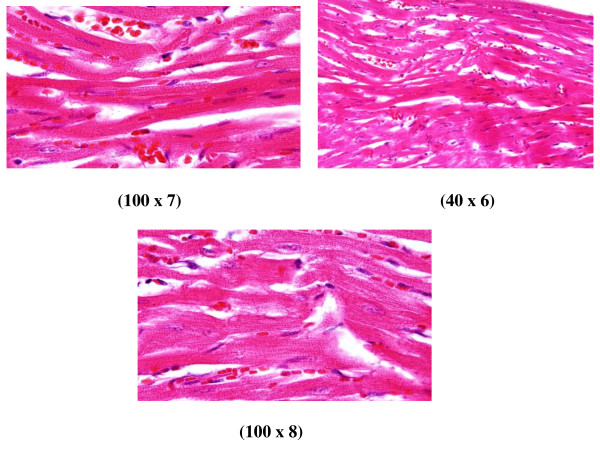
**GNP-treated rat that received 50 μl of 50 nm particles for 7 days demonstrating benign-looking heart muscle, with benign fascicles associated with congested dilated blood vessels and extravasation of red blood cells**.

In comparison with the control group, the following histological alterations were detected in the heart tissue of GNP-treated rats. These histological alterations were observed in Figures 2, 3, 4, 5, 6 and 7. The histological alterations can be summarized as follows:

**1) **Extravasation of red blood cells, a few scattered, lymphocytic infiltrated, normal-looking heart muscle cells (Figures 2 and 5: GNPs-treated rat received 50 μl of 10 nm particles for 3 days).

**2) **More dense hemorrhage and excess extravasation of red blood cells (Figure 3: GNP-treated rat received 50 μl of 10 nm particles for 7 days).

**3) **Scattered extravasation of red blood cells, associated with scattered to collected groups of lymphocytic infiltrations (Figure 4: GNP-treated rat received 50 μl of 20 nm particles for 3 days).

**4) **Extravasation of red blood cells with a few scattered lymphocytic infiltrations (Figure 5: GNP-treated rat received 50 μl of 20 nm particles for 7 days).

**5) **Scattered foci of hemorrhage with extravasation of red blood cells; a few scattered cytoplasmic vacuolization, which may indicate a toxic effect (Figure 6: GNP-treated rat received 50 μl of 50 nm particles for 3 days).

**6) **Benign-looking heart muscle with benign fascicles associated with congested dilated blood vessels and extravasation of red blood cells (Figure 7: GNP-treated rat received 50 μl of 50 nm particles for 7 days).

This infiltration was more prominent after 7 days of administration and in rats receiving 10 and 20 nm GNPs than in those receiving 50 nm GNPs. The appearance of lymphocytic infiltration with extravasation of red blood cells in the heart tissue may suggest that GNPs interfere with the antioxidant defense mechanism, leading to reactive oxygen species (ROS) generation which, in turn, may imitate an inflammatory response. Inflammatory cells infiltration was seen in the portal triads and the perioral zones of GNP-treated rats. The infiltrated cells were mainly lymphocytes and plasma cells [20-23].

GNPs were more strongly oxidizing as evidenced by lipid peroxidation [24,25] as well as decreased neutral red retention time (NRRT) and the number of thiol-containing proteins evident in electrophoretic separations. Cadmium may displace iron or copper from metalloproteins, leading to oxidative stress via the Fenton reaction [26].

It has been reported that 5 nm GNPs cause significantly greater oxidative stress and cytotoxicity effects than larger ones [27-29]. The 5 nm GNPs have been shown to catalyze nitric oxide (NO) production from endogenous S-nitroso adducts with thiol groups in blood serum. NO reacts rapidly with superoxide-producing peroxynitrite (ONOO-), which can interact with lipids, DNA, and proteins via direct oxidative reactions or via indirect radical-mediated damage [28]. ROS production could have resulted from the proportionately high surface area of GNPs used in this investigation [29,30].

There are several possible mechanisms of action for the toxicity of particles, including injury of epithelial tissue [30], inflammation, and oxidative stress response [31,32]. At the cellular level, oxidative stress is considered to be of importance [33,34] and there are nanoparticle-induced oxidative stress responses in keratinocytes, macrophages and blood monocytes after in vitro exposure [35,36].

NPs are nearly of the same dimensions as some biological molecules, such as proteins and nucleic acids. Many of these biomolecules consist of long macromolecular chains that are folded and shaped by cooperative and weak interactions between side groups. The GNPs may intrude into these complex folded structures.

GNPs activate the phagocytic activity of the sinusoidal cells by increasing the number of Kupffer cells to aid removal of accumulated GNPs where lysosomes are involved in the intracellular breakdown into small metabolic products. The produced Kupffer cells hyperplasia might be correlated with the amount of injury to the hepatic tissue induced by GNP intoxication and represent a defense mechanism for detoxification. Kupffer cell hyperplasia contributes to hepatic oxidative stress [37].

The scattered cytoplasmic vacuolization may indicate a toxicity effect exhibited as a result of disturbances to membrane function, which leads to massive influxes of water and Na^+ ^due to GNP effects. Cellular swelling may be accompanied by leakage of lysosomal hydrolytic enzymes that lead to cytoplasmic degeneration and macromolecular crowding [38]. The vacuolated cytoplasm of the hepatocytes of the GNPs treated rats might indicate acute and subacute liver injury induced by these NPs. Variable nuclei sizes were observed in some hepatocytes. This change became apparent after 7 days of 50 nm GNPs administration [20-23].

Fatty change was observed in some swelling hepatocytes of rats exposed to 100 μl of 10 nm GNPs and to a lesser extent in those exposed to larger particles. This hepatic liposis was more prominent in rats exposed to GNPs for 7 days than those receiving the treatment for 3 days [20-23]. Hepatocyte fatty change may be due to lipid peroxidation that leads to rough endoplasmic damage and detachment of the cytoplasmic lipoprotein, which indicate abnormal fat metabolism [22,25-27]. The observed abnormal retention of lipids by hepatocytes induced by GNPs may indicate toxic injury to the liver in the form of hepatocyte liposis by these particles [20-23].

The rats receiving 10 and 20 nm GNPs showed hemorrhage and excess extravasation of red blood cells. Less disruption was observed in rats exposed to 50 nm GNPs, while more damage was detected after 7 days than after 3 days of GNP exposure. This change may indicate heart muscle damage and congestion by GNP exposure.

In agreement with bulk surface chemistry, metallic NPs have considerable chemical reactivity, while ionic crystal NPs have been observed to accumulate protein layers when exposed to the cytoplasm or in the lymphatic fluid. This protein layer is possibly involved in the interaction of the nanoparticle with the cellular system.

None of the above changes were observed in the heart muscle of any member of the control group.

The interaction of NPs with living systems is also affected by the characteristic dimensions. As noted above, GNPs of smaller size may penetrate biomolecules, a situation not possible with larger GNPs. It has been reported that inhaled NPs reach the blood and may reach other target sites such as the liver, heart or blood cells [39,40].

Reduction in size results in an enormous increase in surface to volume ratio, so relatively larger numbers of molecules are present on the surface, thus enhancing intrinsic toxicity [41,42]. This may be one of the reasons that smaller GNPs are generally more toxic than larger particles with the same insoluble material when compared on a mass dose basis.

With several different NPs (polyvinyl chloride, TiO2, SiO2, Co, Ni), only Co induced toxicity in endothelial cells, which was accompanied by the production of the pro-inflammatory cytokine interleukin 8 (IL8) [43]. The Cd, Ni and Pb concentrations significantly increased in blood and all tissues of rats after the intraperitoneal administration of 10, 20 and 50 nm GNPs compared with the control, while different changes were observed with the Co concentrations in blood and several tissues of rats. Cobalt concentrations significantly increased with 20 nm GNPs in blood and kidney tissue of rats compared with the control, while it significantly decreased in heart, lung and liver tissues of rats. 10, 20 and 50 nm GNPs may be an effective inducer of oxidative stress in blood and all tissues of rats compared with the control [44].

The present study demonstrates that the inflammation produced in the heart tissue and other tissues/or organs [20-23] was more prominent with smaller GNPs, which induced greater affects, and was related to the time of exposure to GNPs. It has been reported that smaller GNPs cause significantly greater oxidative stress and cytotoxicity effects than larger ones [25-27].

In the present study we did not measure the GNP concentration in urine or feces but this aspect will be taken into consideration in further experiments.

## Conclusions

Histological alterations induced in heart tissue, as shown in the results of the present work, could be an indication of congestion and disarray of heart muscle due to GNP toxicity through an inability to deal with accumulated residues resulting from metabolic and structural disturbances caused by these particles. It was concluded that these alterations are size-dependent, with smaller particles inducing greater damage, with relation to time of exposure to GNPs.

In comparison with the respective control rats, exposure to GNP doses produced heart muscle disarray, with a few scattered chronic inflammatory cells infiltrated by small lymphocytes, foci of hemorrhage with extravasation of red blood cells, some scattered cytoplasmic vacuolization and congested and dilated blood vessels. None of the above alterations were observed in the heart muscle of any member of the control group. The alterations induced by intraperitoneal administration of GNPs were size-dependent, with smaller ones inducing greater affects, and were related to the time of exposure to GNPs.

One mechanism of toxicity of NPs is likely to be induction of ROS and the consequent oxidative stress in cells and organs. The appearance of a few chronic inflammatory cells infiltrated by small lymphocytes, foci of hemorrhage with extravasation of red blood cells, some scattered cytoplasmic vacuolization and congested and dilated blood vessels may suggest that GNPs interact with proteins and enzymes of the hepatic tissue, interfering with the antioxidant defense mechanism and leading to ROS generation. This, in turn, may induce stress in the hepatocytes, causing them to undergo necrosis.

The interaction of GNPs with proteins and various cell types should be considered as part of toxicological evaluation. Additional experiments related to plasma, tissues cytokine, antioxidant defense mechanism, lipid peroxidation, histomorphologcal and ultrastructure will be performed to identify and understand the toxicity and the potential use of GNPs as therapeutic and diagnostic tools.

## Competing interests

The author declares that he has no competing interests.

## Authors' contributions

AMAK analyzed data, interpreted and wrote the final draft of this manuscript. The animal model used in this study was obtained from the Laboratory Animal Center (College of Pharmacy, King Saud University, Saudi Arabia). AMAK conceived the study and its design and obtained research grants for this study. The author read and approved the final manuscript.
